# Effects of continuity of care on health outcomes among patients with diabetes mellitus and/or hypertension: a systematic review

**DOI:** 10.1186/s12875-021-01493-x

**Published:** 2021-07-03

**Authors:** Kam-Suen Chan, Eric Yuk-Fai Wan, Weng-Yee Chin, Will Ho-Gi Cheng, Margaret Kay Ho, Esther Yee-Tak Yu, Cindy Lo-Kuen Lam

**Affiliations:** 1 Department of Family Medicine and Primary Care, The University of Hong Kong, 3/F Ap Lei Chau Clinic, 161 Main Street, Ap Lei Chau, Hong Kong, China; 2 Department of Pharmacology and Pharmacy, The University of Hong Kong, Hong Kong, China

**Keywords:** Continuity of care, Diabetes mellitus, Hypertension, Mortality, Hospitalisation, Accident and emergency attendance

## Abstract

**Background:**

The rising prevalence of non-communicable diseases (NCDs) such as diabetes mellitus (DM) and hypertension (HT) has placed a tremendous burden on healthcare systems around the world, resulting in a call for more effective service delivery models. Better continuity of care (CoC) has been associated with improved health outcomes. This review examines the association between CoC and health outcomes in patients with DM and/or HT.

**Methods:**

This was a systematic review with searches carried out on 13 March 2021 through PubMed, Embase, MEDLINE and CINAHL plus, clinical trials registry and bibliography reviews. Eligibility criteria were: published in English; from 2000 onwards; included adult DM and/or HT patients; examined CoC as their main intervention/exposure; and utilised quantifiable outcome measures (categorised into health indicators and service utilisation). The study quality was evaluated with Critical Appraisal Skills Programme (CASP) appraisal checklists.

**Results:**

Initial searching yielded 21,090 results with 42 studies meeting the inclusion criteria. High CoC was associated with reduced hospitalisation (16 out of 18 studies), emergency room attendances (eight out of eight), mortality rate (six out of seven), disease-related complications (seven out of seven), and healthcare expenses (four out of four) but not with blood pressure (two out of 13), lipid profile (one out of six), body mass index (zero out of three). Six out of 12 studies on diabetic outcomes reported significant improvement in haemoglobin A1c by higher CoC. Variations in the classification of continuity of care and outcome definition were identified, making meta-analyses inappropriate. CASP evaluation rated most studies fair in quality, but found insufficient adjustment on confounders, selection bias and short follow-up period were common limitations of current literatures.

**Conclusion:**

There is evidence of a strong association between higher continuity of care and reduced mortality rate, complication risks and health service utilisation among DM and/or HT patients but little to no improvement in various health indicators. Significant methodological heterogeneity in how CoC and patient outcomes are assessed limits the ability for meta-analysis of findings. Further studies comprising sufficient confounding adjustment and standardised definitions are needed to provide stronger evidence of the benefits of CoC on patients with DM and/or HT.

**Supplementary Information:**

The online version contains supplementary material available at 10.1186/s12875-021-01493-x.

## Background

The global prevalence of chronic non-communicable diseases (NCDs), such as diabetes mellitus (DM) and hypertension (HT), is rising rapidly. By 2030, deaths attributable to NCDs are predicted to be 52 million annually, compared to 36 million in 2008 [1]. Population aging and earlier development of NCDs have exacerbated the burden of disease on healthcare systems [2]. As opposed to communicable diseases, management for NCDs is typically long term and requires ongoing healthcare interventions, such as asymptomatic screening for early diagnosis and addressing adherence to long-term medications [3]. Based on the framework for integrated people-centred health services, the World Health Organization recommends the practice of continuity of care in primary healthcare to optimise the management on NCDs such as diabetes or hypertension [4].

Continuity of care, or the quality of care between patients and providers extending over time and beyond illness episodes, is a key pillar in a good primary care system [5]. Specifically, relational continuity refers to “a therapeutic relationship between a patient and one or more providers that spans various healthcare events and results in accumulated knowledge of the patient and care consistent with the patient’s needs” [6]. As providers gain more knowledge about the patient [7], they can tailor medical advice in subsequent consultations. A high level of continuity of care has also been associated with reduced mortality, fewer hospitalisations, lower healthcare expenses, improved medication compliance, and higher patient satisfaction [8–22].

Evaluating the effect of continuity of care on the health indicators of DM and/or HT patients is challenging due to high methodological heterogeneity amongst studies. Two previous reviews examining the effect of continuity of care on general populations highlighted the problems of inconsistent measurement of continuity of care [22, 23]. Both reviews stated that the number of high-quality studies focusing on continuity of care and health outcomes was insufficient. The effect of continuity of care on complication development or key disease monitoring indicators of DM and/or HT patients, including haemoglobin A1c (HbA1c) and blood pressure, remains difficult to interpret as there are conflicting results. To bridge the gap in evidence, this review aims to examine the association and effect of continuity on the management and outcomes for patients with DM and/or HT. Secondary objectives are to investigate the mechanisms behind the relationship, to assess the quality and strength of the existing evidence, and suggest possible considerations for the interpretation and application of these findings.

## Methods

### Literature search

A systematic literature search was conducted on 13 March 2021 using four databases (PubMed, Embase, MEDLINE and CINAHL plus), and one trial register (ClinicalTrial.gov). PubMed, Embase and MEDLINE were chosen as they were the most extensive and commonly used databases in medical field; CHINAHL plus is specialised in publications for nursing and allied health professionals. Both Embase and MEDLINE were searched via Ovid while CINAHL plus was searched via EBSCOhost. Each database was searched separately. To reduce the risk of publication bias, a search was conducted in ClinicalTrial.gov. Citation searches were also performed on relevant articles obtained from the literature search. Four key inclusion criteria were: 1) patients with hypertension and/or diabetes mellitus; 2) continuity of care; 3) health outcomes, including health indicators and service utilisation; and 4) published in English and after year 2000. The full description of the electronic search terms can be found in Additional file 1.

### Eligibility and screening

Inclusion and exclusion criteria were pre-specified and applied during the search. Studies were included if they were experimental or observational studies that satisfied the following criteria: 1) the subjects were adult patients (≥ 18 years old) with a diagnosis of diabetes mellitus and/or hypertension; 2) the effects of receiving a regular source of care were examined as the study intervention; 3) the study outcome of interests were standardised and measurable health outcomes, including DM/HT related complications, hospitalisation, accident & emergency (A&E) attendance, mortality rate, blood pressure, HbA1c, lipid profile, body mass index (BMI) and medical expenditure; and 4) were published in English from 2000 onwards. Exclusion criteria included review studies, qualitative studies, studies that focused on general population instead of on DM/HT patients only, or studies where the full texts were not available (e.g. conference abstracts only). Results from all searches were stored in Endnote X9. After removal of duplicate studies, three reviewers (K.S.C., W.H.G.C. and M.K.H.) independently screened the studies based on their titles and abstracts. All search results were reviewed by at least two reviewers. Disputes regarding eligibility were discussed until a consensus was reached. Full texts of selected studies were retrieved and reviewed independently by all three authors. Studies were included upon agreement of all three reviewers, and in cases of disagreement, arbitration was carried out by a fourth reviewer (E.Y.F.W.).

### Data extraction and quality assessment

Information including settings, study design, study population characteristics, sample size, subjects mean age, length of study, measurement of continuity of care, study outcomes, results, and discussion were extracted and compiled into a Microsoft Excel spreadsheet for analysis. For ease of comparison, outcomes of all included studies were sorted into two categories: i) health indicators and ii) service utilisation. Case Control Study Checklist and the Cohort Study Checklist of Critical Appraisal Skills Programme (CASP) appraisal checklists [24] were used to evaluate the quality and risks of bias of the included studies. This tool assists in the quality appraisal of the studies in three areas: validity, legitimacy, and local applicability of the findings concluded from studies [24]. Feasibility of meta-analysis was assessed based on three criteria i) comparable continuity of care assessment; ii) comparable outcomes; and iii) study quality and analysis feasibility.

## Results

**Fig. 1:**
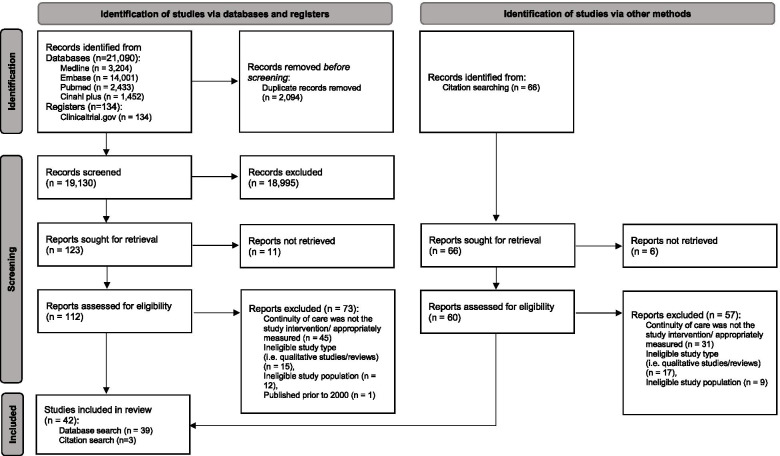
PRISMA 2020 flow diagram of search strategy

The initial search yielded 21,090 studies from the databases and 134 studies from the trial registry. After removing duplicates, screening of the remaining 19,130 studies identified 39 eligible studies. Citation searches of these 39 studies identified 66 potentially relevant articles. Of these, three studies met the eligibility criteria and were included in the review (Fig. 1, Table 1). Among the 42 studies included, seven focused on hypertension [7, 25–30], 32 on diabetes [13, 18, 30–60] and three examined both [10, 61, 62]. Most of them were retrospective cohort studies (31 studies), with a few being prospective cohort (five studies), cross-sectional (four studies), or case–control studies (two studies). These studies were conducted in United States (ten studies), Taiwan (nine studies), South Korea (nine studies), Canada (three studies), Australia (three studies), Malaysia (two studies), United Kingdom (one study), Portugal (one study), the Netherlands (one study), Finland (one study), Chile (one study) and Israel (one study). Most of the studies had a population age mean or median of between 50–70 years. The follow-up periods spanned from less than a year [32, 35, 49] to 17 years [53]; the most common length was 2 to 3 years (16 studies).

**Table 1:** Summary of included studies categorised on study outcomes

**Authors (year)**	**Country**	**Design**	**N**	**Age**^**a**^	**Length (years)**	**CoC measurement**	**CoC cut-off**	**Results**	
								**w/ Improvement**	**w/o improvement**
**I. Health indicators**									
Hanninen, Takala et al. (2001) [31]	Finland	Cross-sectional	DM: 260	< 65	2	Single ph.	Same ph. ≥ 2 years		HbA1c BMI
Overland, Yue et al. (2001) [32]	Australia	Prospective cohort	DM: 479	Single GP: 59.9 (50.7–67.0) Multiple GP: 54.0 (48.7–61.5)	0.5	Single ph.	Single ph.		HbA1c Blood pressure Lipid profile
Parchman and Pugh (2002) [33]	United States	Prospective cohort	DM: 265	58.7 (9.7)	2	CoCI	No cut-off	HbA1c	
Sherina, Teng et al. (2003) [49]	Malaysia	Cross-sectional	DM: 166	59.2	< 1	UPCI	Median		HbA1c
Mainous, Koopman et al. (2004) [34]	United States	Prospective cohort	DM: 1400	No summary	6	Usual ph./site by patient questionnaire	w/ usual ph./site	HbA1c	Blood pressure LDL
Litaker, Ritter et al. (2005) [50]	United States	Retrospective cohort	DM: 1448	No summary	1	Single ph.	Same ph. for 1 year		HbA1c Blood pressure
Fisher, Sloane et al. (2007) [25]	United States	Retrospective cohort	HT: 459	58.9 (14.8)	2	CoCI	0.40 (low/med) 0.67 (med/high)		Blood pressure
Gulliford, Naithani et al. (2007) [35]	United Kingdom	Prospective cohort	DM: 193	65	< 1	Experienced CoC by patient questionnaire	No cut-off		HbA1c Blood pressure BMI
Salzman, Yuen et al. (2006) [30]	United States	Retrospective cohort	HT: 287	≥ 18	3	Single ph.	Same ph. of last 5 visits		Blood pressure
Dearinger, Wilson et al. (2008) [36]	United States	Retrospective cohort	DM: 101	61.8	3	UPCI	0.45 (low/high)	HbA1c	Blood pressure LDL
Younge, Jani et al. (2012) [51]	United States	Retrospective cohort	DM: 484	≥ 18	2	MMCI	Quartiles	HbA1c LDL	Blood pressure
Hanafi, Abdullah et al. (2015) [27]	Malaysia	Retrospective cohort	HT: 1060	62.0 (10.4)	1	UPCI	No cut-off		Blood pressure
Liao, Lin et al. (2015) [52]	Taiwan	Retrospective cohort	DM: 89,428	53.7 (11.1)	10	UPCI (ph. & site)	1.0 in ph. (high) 1.0 in site (high) < 0.7 in both ph. & site (low) Others (med)	Complications (CVD, PVD, renal diseases and others) Hospitalisation Mortality rate	
Lustman, Comaneshter et al. (2016) [42]	Israel	Retrospective cohort	DM: 23,294	High UPCI: 61.1 Low UPCI: 59.7	2	UPCI	0.75 (low/high)	Mortality rate HbA1c Blood pressure	Hospitalisation LDL
Chang, Chien et al. (2018) [53]	Taiwan	Retrospective cohort	DM: 26,063	55.8 (12.0)	17	CoCI	0.43 (low/med) 0.80 (med/high)	Complication (ESRD) Hospitalisation	
Jang, Choy et al. (2018) [54]	South Korea	Retrospective cohort	DM: 3565	No summary	8	CoCI	0.75 (low/high)	Complication (ESRD)	
Khanam, Kitsos et al. (2019) [28]	Australia	Retrospective cohort	HT: 37,425	≥ 18	3.5	HHI	0.5 (low/med) 0.75 (med/high) 1 (max)	Blood pressure	
Kim and Park (2019) [48]	South Korea	Case–control	DM: 55,558	No death: 76.7 (7.0) w/ death: 76.7 (7.1)	12	UPCI (site)	Lowest vs highest by SAS Rank	Mortality rate	
Lee, Chun et al. (2019) [47]	South Korea	Retrospective cohort	DM: 16,806	> 45	12	CoCI	0.75 (low/high)	Complication (thyroid disorder)	
Leniz and Gulliford (2019) [62]	Chile	Cross-sectional	HT: 1252 DM: 418	≥ 15	2	Questionnaire	No cut-off		HbA1c Blood pressure
Nam, Lee et al. (2019) [55]	South Korea	Case–control	DM: 2373	≥ 20	10	CoCI	Median	Complications (CVD, nephropathy and others) Healthcare expense	
Sousa Santos, Tavares Bello et al. (2019) [46]	Portugal	Retrospective cohort	DM: 100	Studied: 69.2 (10.6) Control: 67.2 (10.4)	5	Single ph.	Same ph. ≥ 5 years	HbA1c	Blood pressure BMI LDL
Choi, Choi et al. (2020) [29]	South Korea	Retrospective cohort	HT: 244,187	≥ 20	11	CoCI	0.23, 0.36, 0.56	Complication (CVD)	
**II. Service Utilisation**									
Knight, Dowden et al. (2009) [56]	Canada	Retrospective cohort	DM: 1143	≥ 65	3	CoCI UPCI SECON	0.75	Hospitalisation	
Hong, Kang et al. (2010) [10]	South Korea	Retrospective cohort	HT: 858,927 DM: 268,220	HT: 71.5 (5.0) DM: 70.6 (4.6)	4	CoCI	0.20 (low/med) 0.40 (med/high)	Hospitalisation A&E attendance	
Lin, Huang et al. (2010) [37]	Taiwan	Retrospective cohort	DM: 6476	58.8 (12.7)	5	UPCI	0.47 (low/med) 0.75 (med/high)	Long-term hospitalisation	Short-term hospitalisation
Liu, Doug et al. (2010) [57]	United States	Retrospective cohort	DM: 3873	58.7 (58.3–59.1)	2	FCI (site)	No cut-off	A&E attendance	
Chen and Cheng (2011) [13]	Taiwan	Retrospective cohort	DM: 48,107	60.7 (11.3)	7	CoCI	0.47 (low/med) 0.86 (mid/high)	Hospitalisation A&E attendance Medication expense Healthcare expense	
Robles and Anderson (2011) [7]	United States	Retrospective cohort	HT: 5590	Low CoCI: 76.2 Intermediate: 75.7 High CoCI: 75.9	1	CoCI	0.106 (low/med) 0.236 (med/high)		Medication expense
Worrall and Knight (2011) [38]	Canada	Retrospective cohort	DM: 305	74.3(6.7)	3	UPCI	0.75 (low/high)	Mortality rate Hospitalisation	
Chen, Tseng et al. (2013) [18]	Taiwan	Retrospective cohort	DM: 11,299	55.7(11.3)	7	CoCI	0.22 (low/med) 0.44 (med/high)	Hospitalisation A&E attendance	
Hong and Kang (2013) [39]	South Korea	Retrospective cohort	DM: 68,469	53.6 (12.1)	4	CoCI	0.4, 0.6, 0.8, 1	Mortality rate Hospitalisation Healthcare expense	
Hussey, Schneider et al. (2014) [40]	United States	Retrospective cohort	DM: 166,654	> 65	2	CoCI (ph./site)	No cut-off	Hospitalisation A&E attendance Complications (MI, renal diseases and others) Healthcare expense	
Comino, Islam et al. (2015) [58]	Australia	Retrospective cohort	DM: 20,433	≥ 45	1.5	UPCI	0.80	Hospitalisation	
Cho, Nam et al. (2016) [59]	South Korea	Retrospective cohort	DM: 5163	≥ 20	9	CoCI	0.2, 0.4, 0.6, 0.8, 1	Hospitalisation	
Hsu, Chou et al. (2016) [41]	Taiwan	Retrospective cohort	DM: 3757	No summary	7	CoCI	Low, medium, high (= 1)	A&E attendance	
Nam, Cho et al. (2016) [26]	South Korea	Retrospective cohort	HT: 3,460,700	≥ 20	3	CoCI	0.75 (low/high)	Hospitalisation	
Pu and Chou (2016) [61]	Taiwan	Retrospective cohort	HT: 331,506 DM: 82,181	HT: w/ A&E: 71 No A&E: 66 DM: w/ A&E: 69 No A&E: 65	2	CoCI	HT: 0.46 (low/med) 0.82(med/high) DM: 0.43 (low/med) 0.72(med/high)	A&E attendance	
Van Loenen, Faber et al. (2016) [43]	the Netherlands	Cross-sectional	DM: 45,082	No summary	3	ph. and patient questionnaires	No cut-off		Hospitalisation
Weir, McAlister et al. (2016) [44]	Canada	Prospective cohort	DM: 285,231	53.0 (10.5)	7	UPCI	0.75 (low/high)	Mortality rate Hospitalisation	
Li (2019) [45]	Taiwan	Retrospective cohort	DM: 4007	High CoCI: 61.1 (10.6) Low CoCI: 60.8 (10.5) High UPCI: 61.1 (10.6) Low UPCI: 60.8 (10.5)	3	CoCI UPCI	Median (low/high)	Hospitalisation A&E attendance	Mortality rate
Chen and Cheng (2020) [60]	Taiwan	Retrospective cohort	DM: 57,965	56.3	4	CoCI	Tertiles	Hospitalisation	

*HT* Hypertension, *DM* Diabetes mellitus, *ph.* Physician, *CoC* Continuity of care, *CoCI* Continuity of care index, *UPCI* Usual provider continuity index, *SECON* Sequential continuity index, *MMCI* Modified modified continuity index, *FCI* Fragmentation of care index, *HHI* Herfindahl–Hirschman index, *A&E* Accident and emergency, *HbA1c* Haemoglobin A1c; “Blood pressure” refers to either systolic blood pressure, diastolic blood pressure and a combined target of the two, *BMI* Body mass Index; “Lipid profile” (unless specified) refers to either levels of low density lipoprotein, high density lipoprotein, cholesterol or triglyceride, *LDL* Low-density lipoprotein; “Complications” refers to (but not limited to) onset of cardiovascular disease, end-stage renal disease etc., *ESRD* End-stage renal disease, *CVD* Cardiovascular diseases, *MI* Myocardial infarction, *w/* With, *w/o* Without, *N* Number

^a^Age summary was extracted based on the availability of the information by the following order: mean (SD), median (interquartile range), median, mean or range

Meta-analysis was not performed due to the clear heterogeneity among studies, including differences in assessment methods and cut-off points of continuity of care, inclusion and exclusion criteria of participants and outcomes, as well as adjustment of confounders. There was also a lack of consistency in the reporting of effect size. Missing information such as the number of patients and cases in each group or the exact cut-off points of exposure in some studies made unification of effect size impossible.

### Continuity of care measurement tools

Details of the definitions and formulae of all continuity of care measurement tools used in the reviewed studies are listed in Additional file 2. The two most frequently used continuity of care instruments were the continuity of care index (CoCI) (20 studies) [7, 10, 13, 18, 25, 26, 29, 33, 39–41, 45, 47, 53–56, 59–61] and usual provider continuity index (UPCI) (12 studies) [27, 36–38, 42, 44, 45, 48, 49, 52, 56, 58]. CoCI measures the dispersion of visits of a patient to different providers [63], which is calculated by $\frac{{\sum }_{i=1}^{k}{n}_{i}^{2}-N}{N(N-1)}$, where *k* is the number of providers, ${n}_{i}$ is the number of visits to provider *i* and *N* is the total number of visits of the patient. UPCI measures the proportion of visits given by the most frequently visited provider, i.e. $\frac{{n}_{max}}{N}$, where ${n}_{max}$ is the number of attendances to the most frequently visited provider and *N* is the total number of visits of the patient. Five studies used ‘attendance to a single physician within a period of time’ to define continuity of care [30–32, 46, 50], two studies assessed continuity of care based on the location where medical services were provided, rather than on individual physicians [48, 57], and three examined both [34, 40, 52]. Explanations for why a certain method was used were rarely provided. Two studies used multiple continuity of care calculations and the findings were consistent among different measurements [45, 56]. Among studies that assessed the effect of continuity of care by categorization, there was no consistent definition for cut-off. The most common cut-off used was a UPCI or CoCI of 0.75 (7 studies) to indicate high level of continuity of care [26, 38, 42, 44, 47, 54, 56].

### Study outcomes categories

**Fig. 2:**
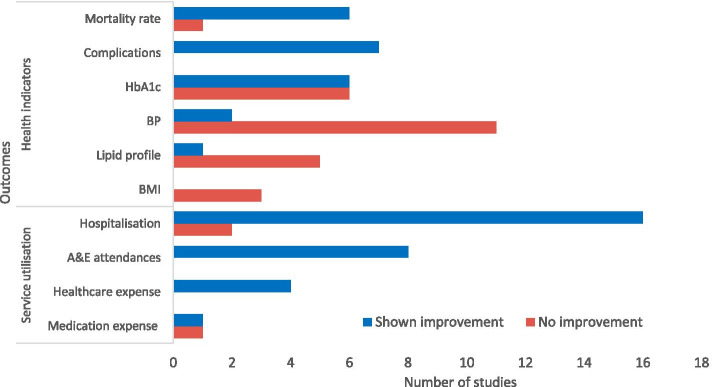
Summary of reported outcomes with/without significant improvement related to continuity of care. Note: For detailed breakdown of study outcomes, please refer to Additional file 4. “Complications” refers to (but not limited to) onset of cardiovascular disease, end-stage renal disease etc.; “BP” refers to either systolic blood pressure, diastolic blood pressure and a combined target of both; HbA1c = Haemoglobin A1c; “Lipid profile” refers to either levels of low-density lipoprotein, high density lipoprotein, cholesterol or triglyceride; BMI = Body mass index; A&E = Accident and emergency

The number of studies showing the effect of continuity of care on various outcome measures is summarised in Fig. 2.

#### Health indicators

Continuity of care was reported by six out of seven studies to reduce mortality [38, 39, 42, 44, 45, 48, 52]. The effect of continuity of care on HbA1c was frequently evaluated in studies focusing on DM patients, but findings were less consistent, with six of the twelve studies showing statistically significant improvements [33, 34, 36, 42, 46, 51]. Continuity of care had little effect on blood pressure, with only two [28, 42] out of 13 studies reported improvements [25, 27, 28, 30, 32, 34–36, 42, 46, 50, 51, 62]. None of the studies showed any improvement in lipid profile [32, 34, 36, 42, 46, 51] or BMI [31, 35, 46]. All seven studies that examined the risk of disease-related complications such as cardiovascular disease, end-stage renal disease found that continuity of care reduced complication risks [29, 40, 47, 52–55].

#### Service utilisation

Hospitalisation and Accident and Emergency (A&E) attendance were the two most frequently studied service utilisation outcomes. Out of 18 studies, 16 reported statistically significant reductions in hospitalisation rates for patients with higher continuity of care and all eight studies that investigated A&E attendance found improvements. Most of the studies measured disease-related service use only [10, 13, 18, 26, 37, 38, 40, 41, 43, 52, 53, 60, 61]. Five studies examined the correlation between continuity of care and all-cause service utilisation [41, 42, 44, 45, 56]. In both cases, increased levels of continuity of care were associated with significant reductions in service use. Four studies showed reduced overall healthcare expenses [13, 39, 40, 55] and one out of two studies reported reduced medication expenses [7, 13].

### Quality of included studies

The quality of the studies was evaluated using the Critical Appraisal Skills Programme (CASP)’s Case Control Study Checklist for Kim et al. and the CASP Cohort Study Checklist for the rest (Additional file 3). One of the most common sources of bias was inadequate accounting for confounding factors, such as socioeconomic status [13, 26, 38, 39, 45, 47, 50] and disease severity and/or comorbidity [38, 40, 61]. Many studies used pre-existing databases for analyses and certain aspects of patient characteristics and/or health-seeking behaviour were not included due to lack of data. There were also potential issues with selection bias and local applicability of findings. Some studies excluded patients who died during the study period or within certain periods following baseline [7, 10, 39, 40, 58] which can bias against patients with greater disease severity. Narrow inclusion criteria reduce the generalisability of the studies. In Worrall and Knight and Hussey et al., only records of fee-for-service were used for analyses [38, 40]. In Litaker et al., patients were recruited from two veteran clinics in the same region [50]. Due to the calculation of proportions, it was quite common for studies using UPCI or CoCI to exclude patients with fewer than three to four attendances [10, 13, 18, 26, 36, 37, 39, 42, 44, 45, 61]. Whether such exclusions may affect the studies’ findings remains unclear. It was also difficult to evaluate whether the length of follow-up was sufficient, since there are no standardised recommendations for the study of continuity of care. Our review found that confounding factors, selection bias, and short follow-up periods were common study limitations.

## Discussion

This review identified studies from a wide range of settings representing a broad range of healthcare systems. The potential influence of different contexts and a diverse range of outcomes were assessed and categorised accordingly to allow for robust comparisons to be made between studies. These categories reflect a logical progression of stages in the management and control of NCDs allowing for a comprehensive synthesis of the current evidence on the effect of continuity of care for patients with diabetes and/or hypertension.

Beneficial effects of continuity of care were reported more consistently for service utilisation, mortality and disease-related complications than for health indicators such as HbA1c, blood pressure. To better understand the potential mechanisms behind the beneficial effect on service utilisation, the principle of how continuity of care comes into play in healthcare provision must be considered. Continuity of care has been described as a three-layered concept that encompasses informational continuity, longitudinal continuity and interpersonal continuity [20]. Interpersonal continuity is the most frequently examined aspect of continuity of care. It refers to the sustained and ongoing caring relationship, which catalyses the delivery of patient-centred healthcare, between the physician and the patient [35, 64]. It also reflects mutual trust and responsibility in this relationship. Healthcare providers with high levels of continuity of care are able to communicate better with their patients and thus have a better knowledge of their patients’ disease history and current situation that might not be included in the patient’s medical records. A study found that higher continuity of care was associated with better quality of care among DM patients, including more HbA1c testing and eye or foot examinations [65]. Therefore, deterioration in patients’ conditions could be less likely to go undetected or untreated. Moreover, continuity of care improves patient satisfaction [35], facilitates higher patient self-care behaviours, compliance and adherence to physicians’ recommendations and regime, which could be the reason for reduced preventable hospital admissions [18, 33, 36].

The reasons behind a lack of significant results among studies in other outcome categories could be due to methodological limitations such as insufficient sample sizes, and use of short-term dynamic outcome measurements. Firstly, studies without significant findings often contained smaller sample sizes [25, 30–33, 36, 46, 51, 62]. Having a small or inadequately powered sample size can affect the validity of results and may mask potential associations between continuity of care and study outcomes. Secondly, biochemical measurements might not be the most reflective or accurate indicators for the effectiveness of continuity of care due to their dynamic nature [34, 36]. Mainous III et al., found a positive association between better continuity of care and improved HbA1c, but not with blood pressure or low-lipoprotein cholesterol among DM patients suggesting the result could be because providers placed a higher priority on achieving glycaemic control for DM patients over other outcomes [34]. As a result, significant associations between glycaemic control and relational continuity of care might be more observable. A similar conclusion was reported by Dearinger et al. [36]. The authors noted that blood pressure is a “dynamic measurement that can be influenced by many factors at any given time”, such as variation among operators which may have contributed to measurement bias [36]. Moreover, blood pressure measurement in the study was performed as part of the patient’s normal consultation, rather than using more meticulous methods, such as ambulatory or home-based measurements, which may impact the level of accuracy in the measurement. This may partially explain why blood pressure was not seen as a significant outcome in association with continuity of care. Both studies highlight the importance of best practice measurements when it comes to evaluating patient health. Careful consideration is needed to discern whether such dynamic outcomes when measured in a study accurately translates to the patient’s health and wellbeing.

Another issue identified in the reviewed studies was a lack of consistent cut-off points among studies that categorised levels of continuity of care into discrete groups. UPCI or CoCI at ≥ 0.75 was a relatively common definition for high continuity of care [26, 37, 38, 42, 44, 47, 54, 56]. However, a number of studies applied other definitions, for example, Robles et al.: CoCI ≥ 0.236 [7]; Dearinger et al.: UPCI ≥ 0.45 [36]; Hong et al.: CoCI ≥ 0.40 [10]; and Chen et al.: CoCI ≥ 0.44 [18]. As there is currently no consensus to what a high degree of continuity of care is, studies often defined their cut-offs based on the distribution of their study population, such as using the median and tertiles. While this might not have a great impact on the overall conclusion for individual studies, it reduces the comparability between studies and may have greater implications for policymaking to improve continuity in real-life settings, as there needs to be evidence-based aims for various health systems to strive towards.

In terms of the quality evaluation of included studies, consideration of confounding factors was one of the biggest challenges. Although most studies had controlled for confounding factors, it was unclear these adjustments were sufficiently comprehensive. Several factors were found to have a significant influence on continuity, such as patient’s perception on continuity, remoteness and characteristics of the patient, the physician or the healthcare organisation [66, 67], which could be potential confounders. Depending on the health system, socio-economic status, which is known to have a significant correlation with health [68], may affect an individual’s ability to continue seeing the same physician which would hence impact their continuity of care. Structure of the healthcare organisation can also have an impact, as continuity of care might be more difficult to sustain in larger group practices [69]. Therefore, it is important to adequately appraise the appropriate confounders to ensure a valid result.

The study context should also be considered in order to understand the nuances of the relationship between continuity of care and patient health outcomes. The studies included in this review took place across several countries, with different healthcare systems. The impact of culture on the physician–patient relationships and/or the actual patient outcomes may be an important confounding factor. For example, Taiwan’s healthcare system offers free consultations with lenient referral practices. This might increase the likelihood of hospital admissions compared to if the study was performed in a country with stricter referral policies [37]. And in many European countries, continuity of care is a well-established concept, emphasized by both healthcare providers and patients [70–72]. These countries generally have strong primary care systems and have implemented various policies to promote continuity of care, such as mandating or providing financial incentives for patients to register with a primary care doctor [73, 74]. This might result in insignificant findings due to low levels of variability in continuity of care among patient populations [43]. While contextual factors do not necessary detract from the findings in these studies, they still need to be taken into consideration when applying the findings to policymaking in other settings.

There were several strengths to this review. Firstly, we studied a wide range of DM/HT-related outcomes. This enabled us to provide a more comprehensive narrative about the effect of relational continuity of care in DM and/or HT patients. Second, we summarised the common characteristics and limitations of existing literature to inform policy recommendations and future research. There were also several limitations. Firstly, no meta-analysis was performed. While we have provided an integrated summary on the associations of continuity of care with various DM/HT related outcomes, without meta-analysis, we are unable to examine the strength of such associations. However, given the heterogeneity, incomplete information and pre-existing biases among the observational studies, results from a meta-analysis would be questionable if not spurious [75]. Second, we did not review the non-English literature which introduces English-language bias. As the implementation and effects of continuity of care may differ across healthcare settings (such as the influence of local health-seeking behaviours), excluding non-English studies can cause selection bias. Third, while we have used a systematic approach to minimize biases, our review protocol lacks prior registration.

One of the challenges encountered in this review was overcoming discrepancies in the definitions of high and low levels continuity. While our review took such classifications as stated in the selected studies, further research should explore how differences in these classifications may affect the resulting conclusions and their statistical significance. Also, as research in this field is still relatively incomplete, there is no consensus on the best way to measure continuity. While this study examined various measures such as CoCI and UPCI, it may be worthwhile to further investigate into the strengths and weaknesses of each measure and how they might influence outcomes.

## Conclusions

This review found strong associations between high continuity of care and reduced healthcare utilisation, mortality rate and complication risk in patients with diabetes and/ or hypertension, but hardly any impact on various health indicators. Heterogeneity in patient selection, confounding adjustment and assessments of continuity of care and outcome variables remain major constraints in current literatures. A more standardised measurement of continuity of care is needed in order to provide more reliable and comparable evidence for its implementation as a healthcare policy.

## Supplementary Information

**Additional file 1.** Full electronic search terms used in literature search.

**Additional file 2.** Definition and formula of continuity of care measurements.

**Additional file 3.** Result post-appraisal by Critical Appraisal Skills Programme (CASP) checklists.

**Additional file 4.** Number studies with significant improvement over the total number studies by each outcome.

## Data Availability

No datasets were generated or analysed in this systematic review. All data extracted and used for the analysis was summarized in Table 1.

## Abbreviations

NCD: Non-communicable diseases
DM: Diabetes mellitus
HT: Hypertension
CoC: Continuity of care
HbA1c: Haemoglobin A1c
A&E: Accident & emergency
CASP: Critical Appraisal Skills Programme
CoCI: Continuity of care index
UPCI: Usual provider continuity index
BMI: Body mass index
LDL: Low-density lipoprotein cholesterol
CVD: Cardiovascular diseases
ESRD: End-stage renal disease
MI: Myocardial infraction
N: Number
SES: Socioeconomic status
