# Supplementary material for: Effects of continuity of care on health outcomes among patients with diabetes mellitus and/or hypertension: a systematic review
Source: BMC Fam Pract. 2021 Jul 3;22:145. doi: 10.1186/s12875-021-01493-x (PMC8254900; doi:10.1186/s12875-021-01493-x)
Supplement: Supplementary file 1 — Additional file 1. Full electronic search terms used in literature search. [file 12875_2021_1493_MOESM1_ESM.docx]

**Additional file 1:** Full electronic search terms used in literature search

Search terms used for Embase <1974 to 2021 Week 09> via Ovid:

1 renovascular hypertension.mp. or exp renovascular hypertension/ or renal hypertension.mp.

2 exp hypertension/ or hypertension.mp.

3 pulmonary hypertension.mp. or exp pulmonary hypertension/

4 portal hypertension.mp. or exp portal hypertension/

5 Diabetes Mellitus.mp. or exp diabetes mellitus/

6 exp non insulin dependent diabetes mellitus/ or Diabetes Mellitus, Type 1.mp. or non insulin dependent diabetes mellitus.mp.

7 exp insulin dependent diabetes mellitus/ or Diabetes Mellitus, Type 2.mp. or insulin dependent diabetes mellitus.mp.

8 Physician-Patient Relations.mp. or exp doctor patient relationship/

9 "Continuity of Patient Care".mp. or exp *"Continuity of Patient Care"/

10 relational continuity.mp.

11 (care adj3 continuum).mp. [mp=title, abstract, heading word, drug trade name, original title, device manufacturer, drug manufacturer, device trade name, keyword, floating subheading word, candidate term word]

12 "longitudinal care".mp.

13 coordinated care.mp.

14 "continuity of care".mp.

15 Patient Care Team.mp.

16 *"Delivery of Health Care"/

17 care continuity.mp.

18 co-ordinated care.mp.

19 care coordination.mp.

20 care co-ordination.mp.

21 mortality.mp.

22 morbidity.mp.

23 health care cost.mp.

24 hospitalization.mp.

25 health service.mp.

26 emergency health service.mp.

27 Community Health Services.mp.

28 Cardiovascular Diseases.mp.

29 Coronary Disease.mp.

30 Myocardial Ischemia.mp.

31 Myocardial Infarction.mp.

32 (Chronic Kidney Failure or Diabetic Nephropathies).mp.

33 Renal Insufficiency.mp.

34 h?emoglobin A1c.mp.

35 lipid profile.mp.

36 blood pressure.mp.

37 body mass index.mp.

38 1 or 2 or 3 or 4 or 5 or 6 or 7

39 8 or 9 or 10 or 11 or 12 or 13 or 14 or 15 or 16 or 17 or 18 or 19 or 20

40 21 or 22 or 23 or 24 or 25 or 26 or 27 or 28 or 29 or 30 or 31 or 32 or 33 or 34 or 35 or 36 or 37

41 38 and 39 and 40

42 limit 41 to english language

43 limit 42 to yr="2000 -Current"

Search terms used for MEDLINE(R) <1946 to March Week 1 2021> via Ovid:

1 Hypertension, Renal.mp. or exp Hypertension, Renal/

2 Hypertension, Renovascular.mp. or exp Hypertension, Renovascular/

3 exp hypertension/ or hypertension.mp.

4 Hypertension, Pulmonary.mp. or exp Hypertension, Pulmonary/

5 Hypertension, Portal.mp. or exp Hypertension, Portal/

6 Diabetes Mellitus.mp. or exp diabetes mellitus/

7 Diabetes Mellitus, Type 1.mp. or exp Diabetes Mellitus, Type 1/

8 Diabetes Mellitus, Type 2.mp. or exp Diabetes Mellitus, Type 2/

9 Physician-Patient Relations.mp. or exp doctor patient relationship/

10 "Continuity of Patient Care".mp. or exp *"Continuity of Patient Care"/

11 relational continuity.mp.

12 (care adj3 continuum).mp. [mp=title, abstract, original title, name of substance word, subject heading word, floating sub-heading word, keyword heading word, organism supplementary concept word, protocol supplementary concept word, rare disease supplementary concept word, unique identifier, synonyms]

13 "longitudinal care".mp.

14 coordinated care.mp.

15 "continuity of care".mp.

16 Patient Care Team.mp.

17 *"Delivery of Health Care"/

18 care continuity.mp.

19 co-ordinated care.mp.

20 care coordination.mp.

21 care co-ordination.mp.

22 mortality.mp.

23 morbidity.mp.

24 health care cost.mp.

25 hospitalization.mp.

26 health service.mp.

27 emergency health service.mp.

28 Community Health Services.mp.

29 Cardiovascular Diseases.mp.

30 Coronary Disease.mp.

31 Myocardial Ischemia.mp.

32 Myocardial Infarction.mp.

33 (Kidney Failure, Chronic or Diabetic Nephropathies).mp.

34 Renal Insufficiency.mp.

35 h?emoglobin A1c.mp.

36 lipid profile.mp.

37 blood pressure.mp.

38 body mass index.mp.

39 1 or 2 or 3 or 4 or 5 or 6 or 7 or 8

40 9 or 10 or 11 or 12 or 13 or 14 or 15 or 16 or 17 or 18 or 19 or 20 or 21

41 22 or 23 or 24 or 25 or 26 or 27 or 28 or 29 or 30 or 31 or 32 or 33 or 34 or 35 or 36 or 37 or 38

42 39 and 40 and 41

43 limit 42 to english language

44 limit 43 to yr="2000 -Current"

Search terms used for CINAHL plus

S1 (MH "Hypertension+") OR "hypertension" OR (MH "Hypertension, Renovascular") OR (MH "Hypertension, Pulmonary+") OR (MH "Hypertension, Renal+") OR (MH "Hypertension, Portal+")

S2 (MH "Diabetes Mellitus+") OR "Diabetes Mellitus" OR (MH "Diabetes Mellitus, Type 2") OR (MH "Diabetes Mellitus, Type 1+")

S3 (MH "Physician-Patient Relations") OR "doctor patient relationship"

S4 (MH "Continuity of Patient Care+") OR "Continuity of Patient Care"

S5 "relational continuity" OR "care continuum" OR "longitudinal care" OR "coordinated care" OR "continuity of care" OR "Patient Care Team" OR "Delivery of Health Care" OR "care continuity" OR "co-ordinated care" OR "care coordination" OR "care co-ordination"

S6 (MH "Mortality") OR "mortality"

S7 (MH "Morbidity") OR "morbidity"

S8 (MH "Health Care Costs") OR "health care cost"

S9 (MH "Hospitalization") OR "hospitalization"

S10 "health service"

S11 (MH "Emergency Medical Services") OR (MH "Emergency Service") OR "emergency health service"

S12 (MH "Community Health Services") OR "Community Health Services"

S13 (MH "Cardiovascular Diseases") OR "Cardiovascular Diseases"

S14 (MH "Coronary Disease") OR "Coronary Disease"

S15 (MH "Myocardial Ischemia") OR "Myocardial Ischemia"

S16 (MH "Myocardial Infarction") OR "Myocardial Infarction"

S17 (MH "Kidney Failure, Chronic") OR "kidney failure, chronic"

S18 (MH "Diabetic Nephropathies") OR "Diabetic Nephropathies"

S19 (MH "Renal Insufficiency") OR "Renal Insufficiency"

S20 (MH "Hemoglobin A, Glycosylated") OR "hemoglobin a1c"

S21 "lipid profile"

S22 (MH "Blood Pressure") OR "blood pressure"

S23 (MH "Body Mass Index") OR "body mass index"

S24 S1 OR S2

S25 S3 OR S4 OR S5

S26 S6 OR S7 OR S8 OR S9 OR S10 OR S11 OR S12 OR S13 OR S14 OR S15 OR S16 OR S17 OR S18 OR S19 OR S20 OR S21 OR S22 OR S23

S27 S24 AND S25 AND S26

Limiters - Published Date: 20000101- 20210231; English Language Expanders - Apply related words; Also search within the full text of the articles; Apply equivalent subjects Search modes - Boolean/Phrase

Search terms used for PubMed:

Search: (((((((((((((((((((((diabetes complications[MeSH Terms])) OR (mortality[MeSH Terms])) OR (morbidity[MeSH Terms])) OR (health care costs[MeSH Terms])) OR (hospitalization[MeSH Terms])) OR (health services[MeSH Terms])) OR (emergency hospital services[MeSH Terms])) OR (community health services[MeSH Terms])) OR (cardiovascular diseases[MeSH Terms])) OR (coronary diseases[MeSH Terms])) OR (myocardial ischemia[MeSH Terms])) OR (myocardial infarction[MeSH Terms])) OR (diabetic nephropathies[MeSH Terms])) OR (renal insufficiencies[MeSH Terms])) OR (kidney failure, chronic[MeSH Terms]))) OR (hemoglobin A1c[MeSH Terms])) OR (lipid profile)) OR (blood pressure[MeSH Terms])) OR (body mass index[MeSH Terms]) ) AND (((((((((((((physician-patient relations[MeSH Terms]) OR continuity of care) OR care continuum) OR relational continuity) OR longitudinal care) OR coordinated care) OR patient care team[MeSH Terms]) OR delivery of health care, integrated[MeSH Terms])) ) OR (care continuity))) OR (co-ordinated care)) OR (care coordination OR (care co-ordination)) AND ((hypertension[MeSH Terms]) OR diabetes mellitus[MeSH Terms]) AND English[Language] AND ("2000/1/1"[Date - Publication] : "3000"[Date - Publication]) Filters: Case Reports, Clinical Conference, Clinical Study, Clinical Trial, Clinical Trial, Phase I, Clinical Trial, Phase II, Clinical Trial, Phase III, Clinical Trial, Phase IV, Controlled Clinical Trial, Observational Study, Randomized Controlled Trial, Humans

Search terms used for Cinicaltrial.gov:

Status: Completed, Suspended, Terminated, Withdrawn, Unknown status Studies

Condition: Hypertension OR diabetes mellitus

Intervention: continuity of care OR "longitudinal care" OR relational continuity OR doctor patient relationship OR coordinated care OR Patient Care Team OR Delivery of Health Care OR care continuity OR co-ordinated care OR care coordination OR care co-ordination

Outcome: Cardiovascular Diseases OR Coronary Disease OR Myocardial Ischemia OR Myocardial Infarction OR chronic kidney failure OR diabetic nephropathies OR Renal Insufficiency OR hemoglobin A1c OR lipid profile OR blood pressure OR body mass index OR mortality OR morbidity OR health care cost OR hospitalization OR health service OR emergency health service OR Community Health Services
