# Supplementary material for: Effects of continuity of care on health outcomes among patients with diabetes mellitus and/or hypertension: a systematic review
Source: BMC Fam Pract. 2021 Jul 3;22:145. doi: 10.1186/s12875-021-01493-x (PMC8254900; doi:10.1186/s12875-021-01493-x)
Supplement: Supplementary file 2 — Additional file 2. Definition and formula of continuity of care measurements. [file 12875_2021_1493_MOESM2_ESM.docx]

**Additional file 2:** Definition and formula of continuity of care measurements

| Measurement | Definition and formula | Number of studies |
| --- | --- | --- |
| Continuity of care index (CoCI) | $COCI= \frac{\sum_{i=1}^{k} n_{i}^{2}-N}{N(N-1)}$  *k* = number of providers  $n_{i}$ = number of visits to provider *i*  *N* = total number of visits of the patient | 20 [7, 10, 13, 18, 25, 26, 29, 33, 39-41, 45, 47, 53-56, 59-61] |
|  |  |  |
| Usual provider continuity index (UPCI) | $UPCI= \frac{n_{max}}{N}$  $n_{max}$ = the number of visits given by the most frequently visited provider  *N* = the total number of visits of the patient | 12 [27, 36-38, 42, 44, 45, 48, 49, 52, 56, 58] |
|  |  |  |
| Single physician/ site | The patient only visited one single physician/site during the measurement period | 5 [30-32, 46, 50] |
|  |  |  |
| Sequential continuity index (SECON) | $SECON=\frac{\varphi i+\ldots+\varphi N-1}{N-1}$  $\varphi$ = 1 when current and the subsequent visits are given by the same provider  $\varphi$ = 0 when current and the subsequent visits are given by the same provider  *N* = the total number of visits of the patient | 1 [56] |
|  |  |  |
| Modified modified continuity index (MMCI) | $MMCI=\frac{1-\frac{k}{N+0.1}}{1-\frac{1}{N+0.1}}$  *k* = number of providers  *N* = total number of visits of the patient | 1 [51] |
|  |  |  |
| Fragmentation of care index (FCI) | $FCI= \frac{n^{2}-\sum_{i=1}^{k} n_{i}^{2}}{N(N-1)}$  *k* = number of providers  $n_{i}$ = number of visits to provider *i*  *N* = total number of visits of the patient | 1 [57] |
|  |  |  |
| Herfindahl-Hirschman index (HHI) | $HHI= \sum_{i=1}^{k} p_{i}^{2}$  $p^{i}$ = proportion of a patient’s visits to provider *i* | 1 [28] |
|  |  |  |
| Experienced continuity of care (ECC) | A 19-item measure on four subdomains: longitudinal, flexible, relational and team and cross-boundary continuity; scores ranged from 0 to 100 [76] | 1 [35] |
|  |  |  |
| Questionnaires (Mainous et al. 2004) | Two questions:  “Is there a particular clinic, health center, doctor’s office, or other place that you usually go to if you are sick, need advice about your health, or for routine care?”  “Is there one particular doctor or health professional you usually see?” | 1 [34] |
|  |  |  |
| Questionnaires (Van Loenen et al. 2016) | Measured on nine scales, including longitudinal continuity and informational continuity. Scale scores range from 0 to 10 | 1 [43] |
|  |  |  |
| Questionnaires (Leniz et al. 2019) | Two questions:  “Do you have your ‘own’ general doctor or family doctor?”  “Do you know the doctor’s name?” | 1 [62] |
