# Supplementary material for: Effects of continuity of care on health outcomes among patients with diabetes mellitus and/or hypertension: a systematic review
Source: BMC Fam Pract. 2021 Jul 3;22:145. doi: 10.1186/s12875-021-01493-x (PMC8254900; doi:10.1186/s12875-021-01493-x)
Supplement: Supplementary file 3 — Additional file 3. Result post-appraisal by Critical Appraisal Skills Programme (CASP) checklists. [file 12875_2021_1493_MOESM3_ESM.docx]

**Additional file 3:** Result post-appraisal by Critical Appraisal Skills Programme (CASP) checklists

|  | CASP checklist used | Q1. | Q2. | Q3. | Q4. | Q5a. | Q5b. | Q6a. | Q6b. | Q7. | Q8. | Q9. | Q10. | Q11. | Q12. |
| --- | --- | --- | --- | --- | --- | --- | --- | --- | --- | --- | --- | --- | --- | --- | --- |
| Hanninen et al. (2001) | CS | + | − | + | + | − | − | n/a | n/a | + | + | + | + | + | + |
| Overland et al. (2001) | CS | + | + | + | + | + | − | + | + | + | + | + | ? | + | + |
| Parchman et al. (2002) | CS | + | + | + | + | + | − | + | ? | + | + | + | ? | + | + |
| Sherina et al. (2003) | CS | + | ? | + | − | − | − | n/a | n/a | + | − | + | − | − | + |
| Mainous et al. (2004) | CS | + | + | − | + | + | + | + | + | + | + | + | + | + | + |
| Litaker et al. (2005) | CS | + | − | + | + | − | + | n/a | n/a | + | + | + | − | − | + |
| Salzman et al.(2006) | CS | + | ? | + | + | − | − | + | + | + | − | + | ? | + | + |
| Fisher et al. (2007) | CS | + | + | + | + | ? | ? | + | + | + | + | + | ? | − | ? |
| Gulliford et al. (2007) | CS | + | + | ? | + | ? | ? | ? | − | + | + | + | + | + | + |
| Dearinger et al. (2008) | CS | + | + | + | + | ? | ? | + | + | + | + | + | ? | + | + |
| Knight et al. (2009) | CS | + | + | + | + | + | + | + | + | + | + | + | + | + | + |
| Hong et al. (2010) | CS | + | + | + | + | + | + | ? | + | + | + | + | + | + | + |
| Lin et al.(2010) | CS | + | + | + | + | + | + | + | + | + | ? | + | ? | + | + |
| Liu et al. (2010) | CS | + | + | + | + | + | + | + | + | + | + | + | + | + | + |
| Chen et al.(2011) | CS | + | + | + | + | + | + | + | + | + | + | + | + | + | + |
| Robels et al.(2011) | CS | + | − | + | + | ? | + | − | − | + | + | ? | − | ? | ? |
| Worral et al. (2011) | CS | + | + | + | + | − | − | ? | ? | + | − | + | ? | + | + |
| Younge et al. (2012) | CS | + | + | + | + | − | − | + | − | + | − | + | + | + | + |
| Chen et al. (2013) | CS | + | ? | + | + | + | + | + | + | + | + | + | ? | + | + |
| Hong et al. (2013) | CS | + | + | + | + | + | + | ? | + | + | + | + | + | + | + |
| Hussey et al.(2014) | CS | + | − | + | + | − | − | ? | ? | + | + | + | ? | + | + |
| Comino et al. (2015) | CS | + | + | + | + | + | + | − | − | + | + | + | + | + | + |
| Hanafi et al (2015) | CS | + | ? | + | + | − | − | − | − | + | + | ? | ? | − | + |
| Liao et al. (2015) | CS | + | + | + | + | + | + | + | + | + | + | + | + | + | + |
| Cho et al. (2016) | CS | + | + | + | + | + | + | + | + | + | + | + | + | + | + |
| Hsu et al. (2016) | CS | + | + | + | + | + | + | ? | + | + | + | + | ? | ? | + |
| Lustman et al. (2016) | CS | + | + | + | + | − | ? | + | ? | + | + | + | − | + | + |
| Nam et al. (2016) | CS | + | + | + | + | + | + | + | ? | + | + | + | − | + | + |
| Pu et al. (2016) | CS | + | + | + | + | − | ? | + | + | + | + | + | + | + | + |
| Van Loenen et al. (2016) | CS | + | − | ? | + | − | − | n/a | n/a | + | + | − | − | − | − |
| Weir et al. (2016) | CS | + | + | + | + | + | + | + | + | + | + | + | + | + | + |
| Chang et al. (2018) | CS | + | + | + | + | + | + | + | + | + | + | + | + | + | + |
| Jang et al. (2018) | CS | + | + | + | + | + | + | + | + | + | + | + | + | + | + |
| Khanam et al.(2019) | CS | + | + | ? | + | + | + | + | + | + | + | + | + | + | + |
| Kim et al. (2019) | CCS | + | + | + | + | + | n/a | + | + | + | + | + | − | + | n/a |
| Lee et al. (2019) | CS | + | + | + | + | − | ? | + | ? | + | + | + | − | + | + |
| Leniz et al. (2019) | CS | + | ? | ? | + | − | + | − | − | + | + | + | ? | + | + |
| Li. (2019) | CS | + | + | + | + | − | ? | + | − | + | + | + | + | + | + |
| Nam et al. (2019) | CS | + | + | + | + | + | + | + | + | + | + | + | + | + | + |
| Sousa Santos et al. (2019) | CS | + | + | + | + | − | − | − | − | + | + | + | − | + | + |
| Chen et al. (2020) | CS | + | + | + | + | + | + | + | + | + | + | + | + | + | + |
| Choi et al. (2020) | CS | + | + | + | + | + | + | + | + | + | + | + | + | ? | + |

Note = “CS” refers to CASP Cohort Study Checklist: <https://casp-uk.b-cdn.net/wp-content/uploads/2018/03/CASP-Cohort-Study-Checklist-2018_fillable_form.pdf>; “CCS” refers to CASP Case Control Study Checklist: <https://casp-uk.b-cdn.net/wp-content/uploads/2018/03/CASP-Case-Control-Study-Checklist-2018_fillable_form.pdf>; “+” refers to “yes”, “-” refers to “no”, “?” refers to “can’t tell” on CASP checklists
