# Supplementary material for: Effects of continuity of care on health outcomes among patients with diabetes mellitus and/or hypertension: a systematic review
Source: BMC Fam Pract. 2021 Jul 3;22:145. doi: 10.1186/s12875-021-01493-x (PMC8254900; doi:10.1186/s12875-021-01493-x)
Supplement: Supplementary file 4 — Additional file 4. Number studies with significant improvement over the total number studies by each outcome. [file 12875_2021_1493_MOESM4_ESM.docx]

**Additional file 4**: Number studies with significant improvement over the total number studies by each outcome

|  | **HT patients** | **DM patients** | **All studies*** |
| --- | --- | --- | --- |
| **I. Health Indicators** | | | |
| Mortality rate [38, 39, 42, 44, 45, 48, 52] | - | 6 / 7 | 6 / 7 |
| HbA1c [31-36, 42, 46, 49-51, 62] | - | 6 / 12 | 6 / 12 |
| BP [25, 27, 28, 30, 32, 34-36, 42, 46, 50, 51, 62] | 1 / 6 | 1 / 8 | 2 / 13 |
| Lipid profile [32, 34, 36, 42, 46, 51] | - | 1 / 6 | 1 / 6 |
| BMI [31, 35, 46] | - | 0 / 3 | 0 / 3 |
| Complications [29, 40, 47, 52-55] | 1 / 1 | 6 / 6 | 7 / 7 |
| **II. Service Utilisation** | | | |
| Hospitalisation [10, 13, 18, 26, 37-40, 42-45, 52, 53, 56, 58-60] | 2 / 2 | 15 / 17 | 16 / 18 |
| A&E attendances [10, 13, 18, 40, 41, 45, 57, 61] | 2 / 2 | 8 / 8 | 8 / 8 |
| Healthcare expense [13, 39, 40, 55] | - | 4 / 4 | 4 / 4 |
| Medication expense [7, 13] | 0 / 1 | 1 / 1 | 1 / 2 |

Note: * Including to 3 studies that studied both HT and DM patients. HT=Hypertension; DM=Diabetes mellitus; HbA1c=Haemoglobin A1c; “BP” refers to either systolic blood pressure, diastolic blood pressure and a combined target of the two; “Lipid profile” refers to either levels of low-density lipoprotein, high-density lipoprotein, or triglyceride; BMI=Body mass index; “Complications” refers to (but not limited to) onset of cardiovascular diseases, end-stage renal disease, nephropathy, neuropathy, etc.; A&E=Accident and emergency
